# Unveiling the Vital Role of Long Non-Coding RNAs in Cardiac Oxidative Stress, Cell Death, and Fibrosis in Diabetic Cardiomyopathy

**DOI:** 10.3390/antiox11122391

**Published:** 2022-12-01

**Authors:** Yuan Tian, Ziting Gao, Wenyun Liu, Jinjie Li, Xin Jiang, Ying Xin

**Affiliations:** 1 Jilin Provincial Key Laboratory of Radiation Oncology & Therapy, The First Hospital of Jilin University, Changchun 130021, China; 2 Department of Gynecology and Obstetrics, The Second Hospital of Jilin University, Changchun 130041, China; 3 Key Laboratory of Pathobiology, Ministry of Education, Jilin University, Changchun 130021, China; 4 Office of Laboratory Management, Jilin University, Changchun 130012, China; 5 Department of Radiation Oncology, The First Hospital of Jilin University, Changchun 130021, China; 6 National Health Commission Key Laboratory of Radiobiology, School of Public Health, Jilin University, Changchun 130021, China

**Keywords:** diabetic cardiomyopathy, cardiac fibrosis, cardiomyopathy death, oxidative stress, long non-coding RNAs, ceRNA

## Abstract

Diabetes mellitus is a burdensome public health problem. Diabetic cardiomyopathy (DCM) is a major cause of mortality and morbidity in diabetes patients. The pathogenesis of DCM is multifactorial and involves metabolic abnormalities, the accumulation of advanced glycation end products, myocardial cell death, oxidative stress, inflammation, microangiopathy, and cardiac fibrosis. Evidence suggests that various types of cardiomyocyte death act simultaneously as terminal pathways in DCM. Long non-coding RNAs (lncRNAs) are a class of RNA transcripts with lengths greater than 200 nucleotides and no apparent coding potential. Emerging studies have shown the critical role of lncRNAs in the pathogenesis of DCM, along with the development of molecular biology technologies. Therefore, we summarize specific lncRNAs that mainly regulate multiple modes of cardiomyopathy death, oxidative stress, and cardiac fibrosis and provide valuable insights into diagnostic and therapeutic biomarkers and strategies for DCM.

## 1. Introduction

Diabetic cardiomyopathy (DCM), a diabetes-induced microvascular complication, is defined as a heart disease in diabetes patients, which results in a structurally and functionally abnormal myocardium in the absence of hypertension, coronary artery disease, and congenital or valvular heart disorders [1]. Approximately 12% of diabetes patients have DCM, which is the main cause of death [2,3]. The main clinical features of DCM are myocardial remodeling, diastolic and systolic dysfunction, and poor prognosis for diabetes patients, which can ultimately result in clinical heart failure (HF) [2,4]. HF can occur in both type 1 diabetes mellitus (T1DM) and type 2 diabetes mellitus (T2DM), and DCM accounts for 44% of deaths in T1DM patients and 52% of deaths in T2DM patients [5]. DCM development and progression are associated with increased myocardial metabolic abnormalities, myocardial apoptosis, autophagy, pyroptosis and ferroptosis, oxidative stress (OS), inflammation, cardiac fibrosis, and microangiopathy [1,6,7].

Long non-coding RNAs (lncRNAs) are a class of RNA transcripts that are longer than 200 nucleotides and have no apparent coding potential [8]. However, lncRNAs play a critical role in regulating the expression of many genes at the transcriptional, post-transcriptional, and translational levels [8,9]. Recent studies have suggested that lncRNAs extensively regulate the pathophysiology of DCM [10,11,12]. In this review, we provide an overview of the specific lncRNAs that participate in the regulation of myocardial cell death, OS, and cardiac fibrosis in DCM. Additionally, we offer insights into the potential significance of lncRNAs and strategies targeting them for the clinical diagnosis and therapy of DCM.

## 2. Characteristics and Biological Activity of lncRNAs

With the rapid development of RNA sequencing, an increasing number of lncRNAs have been discovered and annotated. According to the NONCODE database (http://www.noncode.org, v6.0, accessed on 15 September 2022), 96,411 and 87,890 lncRNA genes are present in humans and mice, respectively. Knowledge of lncRNAs is limited because their number is twice that of human protein-coding genes. Therefore, widespread attention has been focused on the novel functions of lncRNAs as crucial RNA molecules that regulate the expression of genes at the transcriptional, post-transcriptional, and translational levels [13,14,15].

### 2.1. Classification of lncRNAs

The transcripts of lncRNAs are similar to those of mRNAs, which are usually transcribed by RNA polymerase II, then 5′-capped, spliced, and polyadenylated [16,17]. Based on their genomic localization, the generally accepted categorization of lncRNAs is as follows [18,19,20]: (1) sense; overlapping exons by sharing the same promoter, (2) antisense; overlapping exons of another transcript on the opposite strand, (3) intronic; wholly derived from an intron of a protein-coding gene, (4) intergenic; located in the genomic interval between two protein-coding genes, (5) bidirectional; transcribed from bidirectional promoters in the opposite direction of mRNAs, (6) enhancer; transcribed from an enhancer region of a protein-coding gene, and (7) circular lncRNAs; arising from the splicing of a protein-coding gene and forming a covalently enclosed circular RNA.

### 2.2. Functions of lncRNAs

The functions of lncRNAs are closely related to their subcellular localization, although the underlying mechanisms remain elusive and the same lncRNA may function differently by interacting with distinct functional proteins or target partners in the subcellular microenvironment [21]. In the nucleus, lncRNAs regulate transcriptional programs and gene expression through chromatin interactions and remodeling in a cis- or trans-acting manner [22,23]. They can also establish the spatial organization of the nuclear compartment by acting as decoys or scaffolds that recruit RNA-binding proteins [18,24]. In the cytoplasm, lncRNAs participate in signal transduction pathways, gene translation processes, and the post-translational regulation of gene expression. Some lncRNAs can act as competing endogenous RNAs to sequester miRNAs, thereby preventing the repression of their target lncRNAs [25]. Furthermore, lncRNAs can modulate mRNA translation and stabilization or protein localization to regulate protein post-translational modifications [25,26,27]. With advances in experimental techniques, studies have focused on characterizing the functions of lncRNAs in distinct subcellular compartments, including organelles and macromolecular structures [21]. Approximately 15% of the human mitochondrial transcripts are lncRNAs that are regulated by nuclear-encoded proteins [28]. LncRNAs can regulate the structural and functional homeostasis of mitochondria by transferring from the nucleus to the cytoplasm and targeting mitochondrial effector proteins [29,30]. Phase-separation bodies include nuclear speckles, paraspeckles in the nucleus, p-bodies, and stress granules in the cytoplasm. LncRNAs that accumulate in nuclear speckles and paraspeckles regulate pre-mRNA splicing or play a role in the formational and structural integrity of paraspeckles [31,32]. Collectively, lncRNAs play multifaceted roles in different subcellular compartments and in cellular homeostasis.

## 3. The Pathogenesis of Diabetic Cardiomyopathy

### 3.1. Morphological and Structural Characteristics of Diabetic Cardiomyopathy

DCM is a severe diabetic cardiovascular complication that is widely recognized as a risk factor for HF. The major abnormalities in all diabetes patients are hyperglycemia, systemic insulin resistance, and impaired cardiac insulin metabolic signaling. In a prospective national survey, higher glucose levels in patients with HF were associated with increased mortality risk. The incidence of HF in diabetes patients was 39%, higher than that in non-diabetes patients (23%) [33]. Even in patients without a clinical diagnosis of diabetes, there is a linear relationship between blood glucose levels and long-term mortality [1]. Another community-based study of 6814 individuals with no initial coronary artery disease showed that the risk of HF was increased with high indices of metabolic syndrome and that fructose consumption, in particular, may aggravate the development of DCM [34]. No symptoms are observed during the early stages of DCM. The earliest manifestations are left ventricular (LV) hypertrophy and decreased LV compliance [35]. After the onset of systolic dysfunction, LV dilation and reduced ejection fraction (EF) eventually degenerate into clinically symptomatic HF [6]. Both types of diabetes mellitus (DM) cause systemic metabolic derangements, including hyperglycemia and dyslipidemia. In T1DM-associated DCM, the main trigger is hyperglycemia, and its main characteristics are cardiomyocyte loss, increased myocardial collagen deposition, and LV remodeling resulting in impaired LV systolic function [7]. Therefore, symptoms of systolic dysfunction are more typical in T1DM patients. The underlying causes of T2DM include hyperinsulinemia and insulin resistance. Concentric LV remodeling and hypertrophy are due to coronary microvascular inflammation and paracrine effects on cardiomyocytes and endothelial cells, increasing ventricular stiffness and promoting diastolic dysfunction in the early stage of DCM [36,37].

Structural and functional abnormalities in the early stages of DCM are characterized by cardiac fibrosis, hypertrophy, and impaired coronary microvascular perfusion [35]. Cardiac fibrosis, which is characterized by the deposition of collagen types I and III in the interstitium between myocardial fibers [38], may emerge prior to ischemic injury. Cardiac hypertrophy is a prominent feature of diabetic myocardium, which results in increased LV mass and wall thickness, accompanied by compromised systolic and diastolic function [39,40]. High glucose levels may contribute to the induction of cardiac hypertrophy in patients with concomitant obesity or insulin resistance [39,41]. DM patients have increased coronary resistance, decreased coronary flow reserve, and decreased myocardial blood volume and flow [35]. The delivery of oxygen and other essential nutrients to the myocardium is then reduced, which exacerbates microvascular impairments [42,43]. With continuous progression, myocardial and microvascular pathological changes become irreversible and more pronounced in the late phase of DCM, including cardiomyocyte apoptosis, sclerotic small coronary vessels, connective tissue crosslinking, and capillary microaneurysms [44].

### 3.2. The Pathophysiological Mechanisms of Diabetic Cardiomyopathy

**Figure 1:**
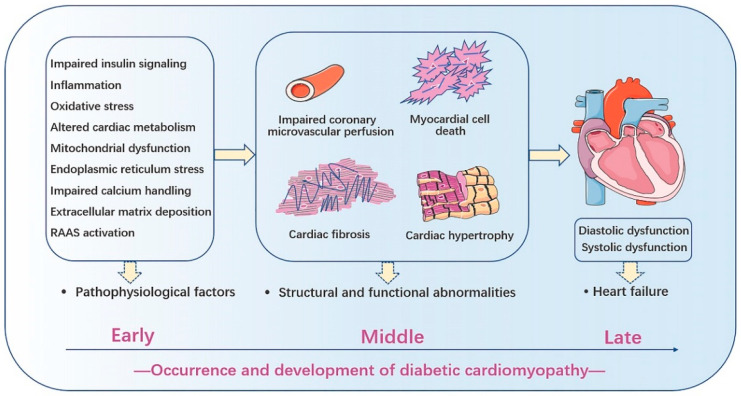
The pathophysiological factors, structural and functional abnormalities in diabetic cardiomyopathy.

The pathophysiological mechanisms of DCM remain unclear, despite an exponential increase in the number of studies in the past few decades. At present, the recognized pathogenic factors of DCM mainly include impaired myocardial insulin signaling and calcium metabolism, systemic glucose and lipid metabolic disorders, elevation in advanced glycation end products (AGEs), mitochondrial dysfunction, accumulation of reactive oxygen species (ROS), activation of inflammation, endoplasmic reticulum (ER) stress, extracellular matrix (ECM) deposition, abnormal coronary microcirculation, endothelial dysfunction, activation of the renin–angiotensin–aldosterone system (RAAS), and cardiac autonomic neuropathy [1,6,7,45]. These pathophysiological changes induce diverse forms of cardiomyocyte death, myocardial fibrosis, hypertrophy, cardiac remodeling, systolic and diastolic dysfunction, and eventually HF [1,6,7] (Figure 1). Hyperglycemia, systemic insulin resistance, and impaired cardiac insulin metabolic signaling are the major clinical abnormalities in all diabetes patients [1]. These mechanisms are involved in the pathogenesis of DCM. Existing clinical observations suggest that the glucose level and dietary consumption in DM patients are risk factors for the development of DCM [33,34]. T1DM is an autoimmune-mediated disease that is mainly insulin deficient, while insulin resistance is more prominent in T2DM. However, DCM induced by both types of DM was characterized by reduced insulin-mediated mitochondrial glucose oxidation [7]. As T2DM progresses and insulin resistance develops, cardiomyocytes take up increased free fatty acids, resulting in impaired mitochondrial fatty acid β-oxidation [46]. Therefore, there is a greater accumulation of toxic lipid metabolites and mitochondrial dysfunction in the hearts of T2DM patients than in those of T1DM patients [44,47].

The pathophysiological factors cause the following structural and functional characteristics of diabetic cardiomyopathy, including myocardial cell death, cardiac fibrosis, cardiac hypertrophy, and impaired coronary microvascular perfusion, finally resulting in heart failure and death.

Lack of insulin or systemic and cardiac insulin resistance induces a decrease in glucose transport and a compensatory increase in insulin production, resulting in hyperinsulinemia and impaired insulin metabolic signaling [44]. Insulin receptor substrate 1 (IRS­1) is an important insulin signaling factor, and increased phosphorylation of IRS-1 results in the activation of the phos­phatidylinositol 3­kinase (PI3K)–protein kinase B (also known as AKT) signal transduction pathway [6]. Under physiological conditions, cardiomyocytes take up glucose via glucose transporter 4 (GLUT4) recruitment to the plasma membrane, which is stimulated by the PI3K/Akt signaling pathway [44]. Therefore, both the deficiency of GLUT4 expression and translocation to the cytomembrane decrease glucose uptake in cardiomyocytes [7]. Activation of PI3K/AKT increases the uptake of free fatty acid (FFA) by promoting the translocation of the FFA transporter CD36 to the plasma membrane. Increased FFA levels activate transcription factors, such as peroxisome proliferator-activated receptor gamma co-activator 1α (PGC-1α) and peroxisome proliferator-activated receptor-α (PPARα), that regulate multiple genes relevant to lipid metabolism [6,7]. Thus, diminished glucose intake, excessive FFA intake, and lipid metabolic disorders disrupt mitochondrial oxidative homeostasis via the tricarboxylic acid (TCA) cycle and β-oxidation [7,48,49]. In DCM, the TCA cycle is disrupted and ATP synthesis is significantly reduced. The reduction in glucose oxidation and glycolysis, accompanied by an increase in fatty acid oxidation, occurs to maintain myocardial substrate utilization [35,39]. Excessive accumulation of fatty acids and lipotoxicity reduce physiological autophagy and lead to impaired myocardial performance [50,51].

Increased generation of reactive nitrogen species (RNS) and ROS caused by mitochondrial dysfunction aggravates ER stress by disrupting protein folding and post-translational modifications [52]. ER stress accelerates membrane instability and Ca^2+^ release from the sarcoplasmic reticulum into the cytosol and also reduces the activity of the sarcoplasmic reticulum calcium pump, which is responsible for Ca^2+^ sequestration during cardiomyocyte relaxation in diastole [6]. The opening of mitochondrial permeability transition pores is induced by Ca^2+^ overload [53], which results in the rapid influx of water and solutes into the mitochondrial matrix and disruption of ATP synthesis [6] .

The generation of mitochondrial ROS that exceeds the endogenous scavenging capacity leads to impaired mitochondrial dynamics, cardiac OS, and inflammation [39]. Persistent OS and hyperglycemia accelerate AGE—RAGE (receptor for AGEs) axis activation [54]. Increased AGE formation and RAGE activation also promote mitochondria-dependent ROS generation, aggravating glucotoxicity and concomitant inflammation [55]. In diabetic settings, activated endothelial cells reduce nitric oxide (NO) levels. The reduction in bioavailable NO impairs coronary microcirculation, increases cardiac stiffness, and impairs relaxation [6]. Normally, NO and endothelium-derived hyperpolarizing factors (EDHFs) released from coronary endothelial cells exert vasodilatory effects and reduce cardiac tissue inflammation [44]. In the early stages of DCM, only NO-induced vasodilation is dysfunctional. However, in later stages, both NO-and EDHF-induced vasodilatations are impaired and promote microvascular dysfunction and inflammation [36,56]. It has also been observed that RAAS activation in a diabetic heart stimulates rapamycin (mTOR)–S6 kinase 1 (S6K1) signaling and impairs myocardial insulin metabolic signaling [57]. Activation of the sympathetic nervous system and RAAS also induces activation of growth and pro-fibrotic signaling pathways, resulting in cardiac fibrosis and diastolic dysfunction [6,44].

**Figure 2:**
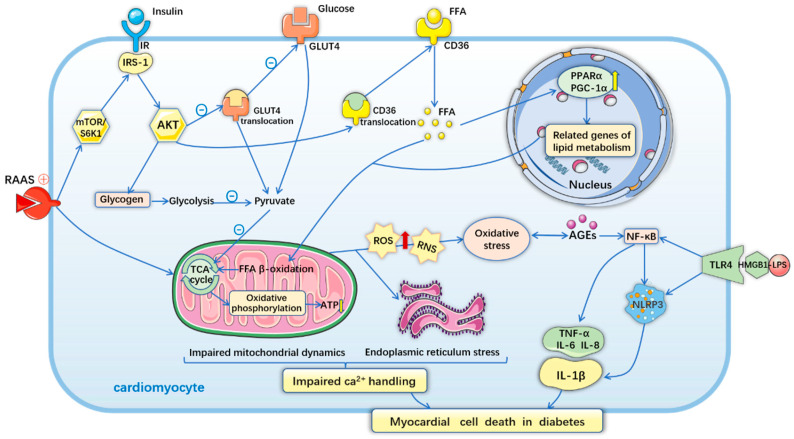
Mechanism of diabetes-induced myocardial cell death in diabetes.

In diabetes, hyperglycemia, hyperlipidemia, and elevated angiotensin II levels activate proinflammatory factors which promote the accumulation and infiltration of cardiac inflammatory cells [7]. It is activated by nuclear factor-κB (NF-κB), a protein complex that controls DNA transcription and inflammatory cytokine production [6]. NF-κB promotes proinflammatory cytokines, including tumor necrosis factor-α, interleukin (IL)-6, and IL-8. It also increases the NLR family pyrin domain-containing 3 (NLRP3) inflammasome assembly, which mediates pro-IL-1β processing and maturation [58], and activates the binding of high mobility group protein B1 (HMGB1) with lipopolysaccharide (LPS) [7]. Toll-like receptor 4 (TLR4) can also induce NLRP3 inflammasome activation, exacerbating inflammation and cell death [59]. The interaction between ROS, ER stress, abnormal calcium handling, and inflammasome activation leads to cardiomyocyte death, which subsequently leads to cardiac hypertrophy, loss of contractility, and cardiac dysfunction (Figure 2). Together, these mechanistic studies highlight the essential role of diverse pathophysiological responses in DCM development (Figure 2). These impaired pathways contribute to the development of DCM and HF by increasing cardiomyocyte death, promoting cardiomyocyte hypertrophy, and impairing cardiomyocyte contractility.

Hyperglycemia, hyperinsulinemia and insulin resistance, activation of the renin–angiotensin–aldosterone system, and mobilization of free fatty acids all can prompt mitochondrial dysfunction, oxidative stress, and endoplasmic reticulum stress. More free fatty acid uptake and oxidation increase the expression of cardiac PPARα and PGC-1α, and then aggravate the reduction of the production of ATP in mitochondria. These cytopathic effects result in intracellular impaired Ca^2+^ handling and Ca^2+^ sensitization increase, further cardiomyocyte death. The systemic glucotoxicity, lipotoxicity, and upregulated angiotensin II production promote proinflammatory processes. HMGB1 binding to LPS increases NLRP3 inflammasome assembling and induces the expression of proinflammatory cytokines, mediating inflammation, and myocardial cell death.

## 4. Role of lncRNAs in Various Types of Cardiomyocyte Death in DCM

**Figure 3:**
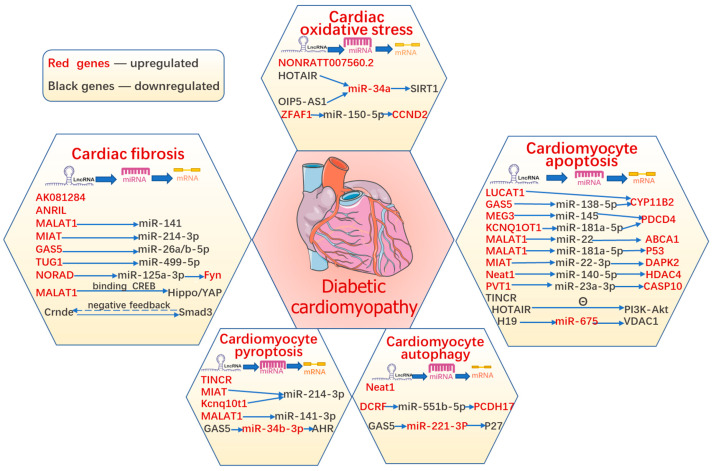
The role of lncRNAs in diabetic cardiomyopathy.

Evidence suggests that various types of cardiomyocyte death simultaneously act as terminal pathways in DCM and eventually accelerate the development of structural and functional impairment in HF, including apoptosis, autophagic cell death, pyroptosis, ferroptosis, and necroptosis. Until now, the suppression of any form of cardiomyocyte death has had a protective function in DCM; however, the regulatory mechanisms of cell death remain unclear and require further clarification. In addition, lncRNAs have been reported to participate in diabetes-induced cardiomyocyte death (Figure 3).

LncRNAs regulate various pathophysiological processes in diabetic cardiomyopathy, including cardiomyocyte apoptosis, pyroptosis, autophagy, and ferroptosis, as well as cardiac oxidative stress and fibrosis. The summary figure shows information on the stud-ies about lncRNAs in DCM.

### 4.1. Role of lncRNAs in Myocardial Apoptosis

Evidence has shown that myocardial apoptosis is a major risk factor in the weakened state of a diabetic heart. Diverse apoptotic inducers and intracellular signaling pathways are activated by diabetic metabolic disturbances [45,60]. Furthermore, apoptotic cell loss promotes cardiac dysfunction and remodeling in DCM [61]. Yin et al. revealed that knockdown of lncRNA lung cancer-associated transcript 1 (LUCAT1) reversed high glucose (HG)-induced cardiomyocyte injury and apoptosis by downregulating cytochrome P450 family 11 subfamily B member 2 (CYP11B2) [62]. In addition, Zhuo et al. confirmed, for the first time, that downregulation of lncRNA growth-arrest specific transcript 5 (GAS5) could reverse cardiomyocyte apoptosis by targeting miR-138-5p and downregulating CYP11B2 expression [63].

Programmed cell death protein 4 (PDCD4) is known to be associated with diabetes [64,65]. Recently, a new study confirmed that knockdown of the lncRNA KCNQ1 Opposite Strand/Antisense Transcript 1 (KCNQ1OT1) downregulated PDCD4 by targeting miR-181a-5p and inhibited myocardial inflammation and apoptosis in DCM [66]. Additionally, lncRNA maternally expressed gene 3 (MEG3) induces apoptosis in AC16 cardiomyocytes under HG conditions by directly binding to miR-145 and upregulating the expression of PDCD4 [67].

The lncRNA metastasis-associated lung adenocarcinoma transcript1 (MALAT1) is widely regarded to have an oncogenic effect. Several studies have shown that lncRNA MALAT1 is involved in the development of cardiovascular disease [68]. MALAT1 knockdown promotes atherosclerotic lesion formation in mice [69]. MALAT1 also regulates endothelial cell function. Silencing MALAT1 disrupts the balance of phenotype switching from proliferative to migratory endothelial cells in vitro and reduces vascular growth in vivo [70]. It is also involved in the pathological process of DCM. MALAT1 expression is significantly upregulated in the cardiac tissue of diabetic rats, and its knockdown attenuates HG-induced cardiomyocyte apoptosis by releasing miR-181a-5p and improving left ventricular function in diabetic rats [71,72]. Enhancer of zeste homolog 2 (EZH2) mediates the methylation of histone H3 at lysine 27 and inhibits the transcription of developmental genes [73]. EZH2 acts as an epigenetic regulator in the progression of cardiac fibrosis through the Smad signaling pathway. It promotes fibroblast differentiation by forming a transcription complex with Smad2 and binding to the promoter region of ACTA2 [74]. The lncRNA nuclear enriched abundant transcript 1 (Neat1) promotes cardiac fibrosis in HF through the increased recruitment of EZH2 to the Smad7 promoter region [75]. MALAT1 mediates cardiomyocyte apoptosis by recruiting EZH2 to the miR-22 promoter and inhibiting its expression in DCM [76]. The myocardial infarction–associated transcript (MIAT) exerts its pro-apoptotic effects by targeting the miR-22-3p/DAPK2 axis in the cultured neonatal cardiomyocytes exposed to HG [77]. MALAT and MIAT were positively correlated among 200 T2DM patients, and high circulating levels of these lncRNAs may be linked to disease severity among T2DM patients [78]. Diabetes-induced cardiomyocyte apoptosis can be mitigated via the activation of nuclear paraspeckle assembly transcript 1 (Neat1)/miR-140-5p/HDAC4 axis [79]. Another competing endogenous RNAs(ceRNA, plasmacytoma variant translocation 1 (PVT1), targets the miR-23a-3p/CASP10 axis in HG-induced cardiomyocyte apoptosis [80]. Silencing the cardiac PVT1 preserves myocardial function in response to DCM.

Some antioxidants, such as lithium chloride, nicorandil, and curcumin, have been shown to alleviate myocardial cell apoptosis and injury in DCM via the PI3K-Akt pathway [81,82,83]. In serum, the lncRNA homeobox transcript antisense RNA (HOTAIR) [84] is downregulated in DM patients and can be used to distinguish them from healthy controls. Furthermore, HOTAIR overexpression improved the viability of HG-treated AC16 cells by activating the PI3K/Akt pathway. Several lncRNAs have been reported to be downregulated in the cardiac tissue and serum of diabetes patients and in rat models. For example, tissue differentiation-inducing non-protein coding RNA (TINCR) expression was significantly lower in the heart and serum of DCM patients than that in diabetes patients without cardiopathy or healthy controls [85]. Furthermore, apoptosis of cardiomyocytes was inhibited by TINCR overexpression under HG conditions. The lncRNA H19 [86] was discovered to upregulate the expression of voltage-dependent anion channel 1 (VDAC1) by targeting miR-675 and inducing mitochondria-mediated apoptosis [87]. The lncRNAs that are mainly involved in the pathogenesis of diabetes-induced myocardial cell apoptosis are summarized in Table 1.

### 4.2. The Role of lncRNAs in Myocardial Autophagy

Autophagy is a protective mechanism that removes damaged proteins, defective organelles, and unwanted cells under normal physiological conditions [88]. It restores cardiac homeostasis by dispensing intracellular lipid droplets, damaged mitochondria, and excessive ROS [89]. Persistent hyperglycemia, dyslipidemia, and inflammation inhibit autophagy in diabetic hearts [90]. Preclinical studies have revealed that the suppression of autophagy deteriorates the development of DCM in T1DM patients [91,92,93]. Glucolipotoxicity in T1DM impairs autophagosomal clearance by reducing lysosomal contents and inducing endoplasmic reticulum and cardiac injury [94]. The lncRNA GAS5 [95] is downregulated in HG-treated H9C2 cells and the myocardium of diabetic rats. In DCM, GAS5 reversed histopathological changes and improved myocardial function by facilitating myocardial autophagy by targeting the miR-221-3p/p27 axis.

However, autophagy is a double-edged sword in DCM [90]. Autophagy hyperactivation associated with impaired insulin signaling can cause the degradation of essential cellular components in T2DM [89,96]. Studies have shown that lncRNAs play pathogenic roles in DCM by accelerating myocardial autophagy. The lncRNA Neat1 exacerbates myocardial ischemia-reperfusion injury by promoting the activation of autophagy and the production of lactate dehydrogenase and serum myocardial enzymes in diabetic rats [97]. In addition, Feng et al. [98] confirmed that the expression of lncRNA DCM-related factor (DCRF) is upregulated in the myocardium of diabetic rats. Furthermore, DCRF knockdown suppressed autophagy activation via targeting the miR-551b-5p/PCDH17 axis in cardiomyocytes treated with HG and improved cardiac function in diabetic rats.

### 4.3. The Role of lncRNAs in Inflammasome-Mediated Myocardial Pyroptosis

Pyroptosis is a type of programmed cell death that is often caused by proinflammatory factors and is characterized by pore formation, disruption of the plasma membrane, and cell swelling [99]. Pathogen- and damage-associated molecules are recognized by NLRP3 during pyroptosis, resulting in the activation of caspsase-1 [45]. Subsequently, caspsase-1 promotes the release of cell contents and inflammatory factors after the cleavage of downstream gasdermin D.

The lncRNAs KCNQ1OT1 and MIAT are upregulated in DM patients, diabetic mice, and high glucose-stimulated cardiomyocytes [100,101,102]. Silencing KCNQ1OT1 or MIAT alleviates myocardial pyroptosis and cytoskeletal structural abnormalities. MiR-214-3p is reduced in the serum of diabetes patients [101]. KCNQOT1 and MIAT also improve cardiac function by sponging miR-214-3p and decreasing caspase-1 expression in DCM [100,101,102]. MALAT1 promotes HG-induced H9C2 pyroptosis by targeting miR-141-3p [103]. The lncRNA TINCR can aggravate pyroptosis in DCM by stabilizing NLRP3 mRNA. METTL14 is a component of N6-methyladenosine (m6A) that downregulates lncRNA TINCR by increasing its m6A methylation level [104]. Therefore, METTL14 suppresses diabetes-induced myocardial pyroptosis by downregulating TINCR expression.

Xu et al. found that lncRNA GAS5 was significantly repressed in the cardiac tissue of diabetic rats; GAS5 overexpression suppressed caspase-1 activity, LDH release, and IL-1β and IL-18 levels in HG-treated cardiac muscle cells (HL-1 cells). They showed that GAS5 sponges miR-34b-3p to enhance aryl hydrocarbon receptor (AHR) expression [105]. AHR is a negative regulator of the NLRP3 inflammasome that binds to the xenobiotic response element in the NLRP3 promoter [106]. The discovery of these specific lncRNAs and their downstream targets may provide potential intervention targets for pyroptosis in DCM. Table 2 summarizes the lncRNAs that participate in the regulation of myocardial autophagy and pyroptosis in DCM.

## 5. Role of lncRNAs in Oxidative Stress in DCM

Hyperglycemia can induce excess ROS generation through AGEs, the polyol pathway, and de novo synthesis of triose metabolism [107]. Significantly increased ROS levels are a pathological feature of DCM [108] and can overwhelm their removal mechanisms, leading to OS [7]. In DCM, the unbalanced redox state aggravates irreversible damage and death of cardiomyocytes, ultimately leading to cardiac dysfunction [109]. Studies have confirmed that antioxidant interventions to scavenge ROS inhibit or prevent cardiac dysfunction in diabetic animal models [110,111,112]. Recent evidence suggests that targeting OS using lncRNAs may be a promising approach for DCM. Yu et al. detected the expression of lncRNAs in HG-treated cardiomyocytes and identified the lncRNA NONRATT007560.2 as one of the top three upregulated lncRNAs [113]. Furthermore, inhibition of NONRATT007560.2 lowered the generation of ROS in HG-treated cardiomyocytes, suggesting NONRATT007560.2 can inhibit diabetes-induced myocardial OS.

Sirtuin 1 (SIRT1) is a redox-sensitive enzyme that appears to improve DCM by targeting cellular factors and increasing stress resistance [114]. SIRT1 upregulation attenuated ER stress-induced cardiomyocyte apoptosis [115]. The lncRNAs OIP5-AS1 (Opa-interacting protein 5-antisense transcript 1) and HOTAIR were both significantly decreased in DCM [116,117]. Overexpression of HOTAIR or OIP5-AS1 improves cardiomyocyte viability and alleviates OS through the miR-34a/SIRT1 axis; therefore, they may be new therapeutic targets for DCM.

In 2012, Dixon [118] first proposed a new concept of regulated cell death called ferroptosis, which is caused by the intracellular deposition of iron and lipid peroxide after excessive accumulation of ROS. In DCM, the essential factor in ferroptosis is OS injury [118]. In vivo, the activation of nuclear factor erythroid 2-related factor 2 (NRF2) by sulforaphane (SFN) alleviated the progression of DCM by inhibiting myocardial cell ferroptosis [119]. NRF2 plays a critical role in regulating the cellular antioxidant response by controlling the expression of many genes that counteract the effects of OS. The lncRNA zinc finger antisense 1 (ZFAS1) was shown to promote cardiomyocyte ferroptosis in DCM by sponging miR-150-5p and downregulating cyclin D2 (CCND2), which contributes to myocardial repair by regulating the cell cycle [120]. The regulation of lncRNAs during OS in DCM is summarized in Table 3. The roles of lncRNAs in OS in DCM are summarized in Figure 3.

## 6. Role of lncRNAs in Diabetes-Induced Cardiac Fibrosis

Cardiac fibrosis is an important cause of cardiac dysfunction in DCM patients. Abnormally elevated ECM deposition, in particular collagen, increases myocardial stiffness and results in ventricular remodeling and dysfunction of LV relaxation and contraction [88]. Hyperglycemia activates the matrix-synthesis program in cardiac fibroblasts by the stimulation of transforming growth factor β (TGF-β) cascades, resulting in the accumulation of AGEs and AGE-mediated fibroblasts [121,122,123]. Rapid activation of TGF-β exerts a broad range of direct effects on cardiac fibroblasts (CFs) [124], including the activation of downstream Smad-dependent signaling cascades, the induction of myofibroblast conversion, and the accumulation of ECM [125,126]. Melatonin, a hormone produced by the pineal gland, has an anti-fibrotic effect on DCM pathogenesis. Melatonin can inhibit TGF-β1/Smad2/3 signaling and NLRP3 inflammasome activation, which can be mediated via the MALAT1/miR-141 axis [127]. The lncRNA colorectal neoplasia differentially expressed (Crnde) is a cardiac-specific and CF-enriched lncRNA that has been found to be negatively correlated with the cardiac fibrosis marker genes in 376 human heart tissues [128]. Overexpression of Crnde attenuated myofibroblast differentiation and cardiac fibrosis in DCM mice by inhibiting the transcriptional activation of Smad3. Interestingly, Smad3 also transcriptionally activated Crnde expression, indicating the existence of a delicate Smad3-Crnde negative feedback.

Inflammatory cytokines directly stimulate the recruitment and activation of lymphocytes and macrophages and promote the pathogenesis of cardiac fibrosis. Myriad proinflammatory cytokines and chemokines are secreted by proinflammatory macrophages in injured cardiac tissues [129]. Their crosstalk with fibroblasts promotes fibroblast differentiation into myofibroblasts, exacerbating extracellular matrix deposition [130]. TGF-β stimulates NLRP3 expression and activates α-SMA, thereby promoting myofibroblast differentiation [131]. IL-17 is a proinflammatory cytokine secreted by activated CD4+ T cells [132]. IL-17 accelerates the production of IL-6 in cardiac fibroblasts, leading to myofibroblasts [133]. The expression of IL-17 and lncRNA MIAT is significantly upregulated in the serum of diabetes patients [132]. MIAT inhibits IL-17 production by specifically attenuating miR-214-3p in primary cardiac fibroblasts. Consequently, decreased IL-17 expression alleviates the onset of cardiac fibrosis and improves cardiac contractility. Ablation of IL-17 improved cardiac function and alleviated cardiac interstitial fibrosis by inhibiting the lncRNA AK081284 in diabetic mice [134].

Vascular endothelial growth factor (VEGF) is activated by sustained metabolic and hemodynamic perturbations in diabetes. VEGF mediates ECM deposition and aggravates cardiac fibrosis by upregulating the pro-fibrotic growth factors TGFβ1 and connective tissue growth factor (CTGF) [135]. The lncRNA ANRIL (antisense non-coding RNA in the INK4 locus) is a recruiter of the polycomb repressive complex (PRC) that facilitates the alteration of chromatin structure [136]. Thomas et al. discovered that ANRIL promotes the synthesis of ECM and VEGF via epigenetic upregulation of EZH2 and the histone acetylator p300, deteriorating cardiac fibrosis in diabetic hearts [137].

The Hippo pathway can negatively regulate the transcriptional coactivators Yes-associated protein (YAP) and transcriptional coactivator with PDZ-binding motif (TAZ), through the activation and phosphorylation of large tumor suppressor (LATS)1/2 kinases [138]. Phosphorylation of LATS1 promotes YAP/TAZ nuclear export and abrogates transcriptional effects in the nucleus [124]. YAP/TAZ is implicated in fibrotic actions by driving fibrosis-related target gene expression in the nucleus, accentuating TGF-β-driven activation of Smad2/3, and stimulating fibroblast proliferation [124,139]. Emerging evidence suggests that YAP/TAZ also affects cardiac fibrosis [140,141]. In HG CFs, MALAT1 and YAP expression in the nucleus was markedly increased and the phosphorylation of LATS1 was decreased [142]. MALAT1 positively regulates YAP by binding to cAMP-responsive element-binding protein (CREB). Furthermore, Liu et al. confirmed that MALAT1 knockdown alleviated collagen accumulation and diabetic fibrosis through the Hippo pathway/YAP signaling pathway [142].

The lncRNAs, TUG1 (taurine upregulated gene 1), NORAD (non-coding RNA activated by DNA damage), and GAS5 are upregulated in the myocardial tissues of diabetic mice [143,144,145]. These lncRNAs exacerbate cardiac fibrosis by negatively regulating miR-499-5p, miR-125a-3p, and miR-26a/b-5p [143,144,145]. Table 3 summarizes the lncRNAs implicated in the pathogenesis of cardiac fibrosis in DCM. Figure 3 summarizes the vital roles of lncRNAs in cardiac fibrosis of DCM.

**Table 3:** LncRNAs implicated in the pathogenesis of oxidative stress and cardiac fibrosis in DCM.

LncRNAs	Experimental Model	Target Genes	Expression	Mechanism Involved	References
**Oxidative Stress**					
NONRATT007560.2	HG-treated primary culture of neonatal cardiomyocytes		upregulated	inhibition of NONRATT007560.2 abated the formation of ROS	[113]
HOTAIR	STZ-induced diabetic rat model and HG-treated H9c2 cells	miR-34a	downregulated	HOTAIR protected against DCM via activation of the SIRT1 expression by sponging miR-34a	[116]
OIP5-AS1	HG-treated H9c2 cells	miR-34a	downregulated	OIP5-AS1 overexpression promoted viability and inhibits high glucose-induced oxidative stress of cardiomyocytes by targeting miRNA-34a/SIRT1 Axis	[117]
ZFAS1	STZ-induced diabetic mouse model and primary culture of neonatal cardiomyocytes	miR-150-5p	upregulated	inhibition of ZFAS1 attenuated ferroptosis by sponging miR-150-5p and activating CCND2 against DCM	[120]
**Cardiac Fibrosis**					
ZFAS1	STZ-induced diabetic mouse model and primary culture of neonatal cardiomyocytes	miR-150-5p	upregulated	inhibition of ZFAS1 attenuated ferroptosis by sponging miR-150-5p and activating CCND2 against DCM	
MALAT1	STZ-induced diabetic mice model and HG-treated primary culture of neonatal CFs	miR-141	upregulated	melatonin alleviated cardiac fibrosis via inhibiting MALAT1/miR-141-mediated NLRP3 inflammasome and TGF-β1/Smads signaling	[127]
Crnde	human myocardial biopsies, STZ-induced diabetic mice model, and HG-treated primary culture of neonatal CFs		downregulated	lncRNA Crnde attenuated cardiac fibrosis via Smad3-Crnde negative feedback	[128]
MIAT	Human serum samples, STZ-induced diabetic mice model, and HG-treated primary culture of neonatal CFs	miR-214-3p	upregulated	MIAT inhibited IL-17 production and alleviated the onset of cardiac fibrosis via specific attenuating miR-214-3p	[132]
AK081284	STZ-induced diabetic mice model and HG-treated primary culture of neonatal CFs		upregulated	AK081284 knockdown inhibited the production of collagen I, collagen III, TGFβ1 and α-SMA stimulated by IL-17	[134]
ANRIL	STZ-induced diabetic mice model		upregulated	ANRIL upregulated production of ECM proteins and VEGF via epigenetic upregulating p300 and EZH2	[137]
MALAT1	STZ-induced diabetic mice model and neonatal mouse HG-treated CFs	CREB	upregulated	MALAT1 regulated diabetic cardiac fibroblasts through the Hippo/YAP signaling pathway by binding CREB	[142]
TUG1	STZ-induced diabetic mice model and HG-treated cardiomyocytes	miR-499-5p	upregulated	inhibition of TUG1 protected against DCM-induced diastolic dysfunction by regulating miR-499-5p	[143]
NORAD	subcutaneous injection of angiotensin II (ATII) in db/db mice and HG-treated primary mouse cardiomyocytes	miR-125a-3p/Fyn	upregulated	silencing NORAD mitigated fibrosis and inflammatory responses via the ceRNA network of NORAD/miR-125a-3p/Fyn	[144]
GAS5	STZ-induced diabetic mice model and HG-treated primary culture of neonatal cardiomyocytes	miR-26a/b-5p	upregulated	silencing GAS5 alleviated apoptosis and fibrosis by targeting miR-26a/b-5p	[145]

## 7. Challenges and Potential Strategies of lncRNA Biomarkers for DCM

The pivotal role of lncRNAs in the multiple pathological mechanisms of DCM has been revealed in recent years. Accumulating evidence has demonstrated that lncRNAs are potential biomarkers for DCM, and the great potential of cardiac lncRNA biomarkers in patient plasma and whole blood is promising for diagnostic and prognostic applications. Markedly altered lncRNAs in DCM patients can be used to distinguish them from healthy controls [84,85,132]; these include lncRNA HOTAIR, TINCR, and MIAT. Li et al. evaluated lncRNA expression profiles in hearts from controls and DCM-induced chronic HF patients and identified 313 significantly differentially expressed lncRNAs [146]. Screening and experimental verification proved that lncRNA DCRL was a human-specific lncRNA associated with DCM patients [146].

Specifically, altered levels of lncRNAs in DCM patients can be used to distinguish DCM from other diabetic complications. Zha et al. compared plasma long intergenic non-protein-coding RNA p53-induced transcript (LINC-PINT) levels in 244 T2DM patients and 126 healthy volunteers. This study showed that LINC-PINT is downregulated in patients who develop cardiomyopathy or retinopathy, or both. Upregulation of LINC-PINT expression may inhibit the progression of cardiomyopathy and retinopathy in T2DM patients [147]. Another vital finding of this study was that LINC-PINT increased the viability of AC16 and ARPE-19 cells after treatment with 20 mM D-glucose. This may contribute to the progression of cardiomyopathy and retinopathy in T2DM patients [147]. LINC-PINT was also found to be significantly overexpressed in acute myocardial infarction (AMI). Downregulation of LINC-PINT facilitated miR-208a-3p expression and suppressed JUN protein levels. It then inhibited activation of the MAPK pathway in AMI tissues and thus alleviated AMI [148]. In an eight-year follow-up study, Li et al. detected plasma NKILA (nuclear factor-κB interacting long non-coding RNA) levels in 312 diabetes patients without significant complications. NKILA plasma levels six months before diagnosis were sufficient to distinguish DCM patients from other diabetes patients [149]. The study also found that NKILA overexpression promoted cardiomyocyte apoptosis in vitro [149]. However, plasma NKILA levels were not significantly altered in patients with diabetes and other complications, further implicating the specific involvement of lncRNA NKILA in DCM. NKILA is also defined as a critical repressor that protects the endothelium from inflammatory lesions [150]. In cardiomyocytes, NKILA can upregulate KLF4, an anti-inflammatory atheroprotective regulator in endothelial cells, through NF-κB-mediated DNA methylation and prevent the inflammatory response [150,151].

Systolic and diastolic dysfunction are not easy to diagnose in the early stages of DCM. A study of 48 men with well-controlled T2DM and 12 healthy age-matched volunteers evaluated the potential of lncRNAs as biomarkers of subclinical cardiac abnormalities in T2DM [152]. Long intergenic non-coding RNA predicting cardiac remodeling (LIPCAR) was inversely associated with diastolic function. Smooth muscle and endothelial cell-enriched migration/differentiation-associated long non-coding RNA (SENCR), LIPCAR, and lncRNA MIAT were directly associated with LV mass to LV end-diastolic volume ratio. These lncRNAs can be used as biomarkers for diastolic function and left ventricular remodeling in T2DM patients [152].

The potential of lncRNAs as biomarker candidates, particularly in the early stages of DCM, requires further investigation. It is challenging to detect lncRNAs in small volumes of plasma or whole blood and to study the extracellular release of lncRNAs into circulation. To determine whether lncRNAs could become novel therapeutic targets for DCM, further research on sequence-specific interactions with RNA through RNA interference drugs or antisense oligonucleotides should be conducted.

## 8. Conclusions

In this review, we summarize recent progress in the pivotal role of lncRNAs in the pathogenesis of DCM. Available evidence suggests that lncRNAs are responsible for the regulation of diverse forms of cardiomyocyte death, cardiac OS, and fibrosis in diabetic hearts. They primarily function as ceRNAs to sponge miRNAs and modulate the expression of target genes. However, further research is needed on other mechanisms, such as post-transcriptional processing or interaction with RNA-binding proteins. Recently, clinical studies have evaluated the diagnostic and prognostic value of lncRNAs as independent predictors of DCM. Further research is needed to elucidate the involvement of lncRNAs and to discover potential biomarkers and treatments for DCM.

## Figures and Tables

**Table 1:** Long non-coding RNAs (lncRNAs) involved in the pathogenesis of cardiomyocyte apoptosis in diabetic cardiomyopathy (DCM).

LncRNAs	Experimental Model	Target Genes	Expression	Mechanism Involved	References
LUCAT1	HG-treated AC16 cardiomyocytes	CYP11B2	upregulated	knockdown of LUCAT1 reversed HG-induced cardiomyocyte apoptosis by down-regulating CYP11B2	[62]
GAS5	STZ-induced diabetic mice model and HG-treated AC16 cardiomyocytes	miR-138-5p/CYP11B2	upregulated	down-regulation of GAS5 reversed cardiomyocyte injury and apoptosis by targeting miR-138 to down-regulate CYP11B2	[63]
KCNQ1OT1	STZ-induced diabetic mice and HG-treated human cardiomyocytes	miR-181a-5p	upregulated	KCNQ1OT1 knockdown inhibited myocardial inflammation and cardiomyocyte apoptosis via targeting miR-181a-5p/PDCD4	[66]
MEG3	HG-treated AC16 cardiomyocytes	miR-145/PDCD4	upregulated	MEG3 induced cardiomyocytes apoptosis through directly binding to miR-145 and upregulating the expression of PDCD4	[67]
MALAT1	STZ-induced diabetic rat model		upregulated	knockdown of MALAT1 associated with an improvement in left ventricular function through reducing cardiomyocyte apoptosis	[71]
	STZ-induced diabetic mice model	miR-181a-5p/P53	upregulated	MALAT1 knockdown attenuated high glucose-induced cardiomyocyte apoptosis via releasing miR-181a-5p and inhibiting P53 expression	[72]
	Spontaneously diabetic (db/db) C57BL/Ks mice model and primary culture of neonatal cardiomyocytes	miR-22/ABCA1	upregulated	MALAT1-mediated recruitment of the histone methyltransferase EZH2 to the microRNA-22 promoter leads to cardiomyocyte apoptosis	[76]
MIAT	STZ-induced diabetic rat model and HG-treated primary culture of neonatal cardiomyocytes	miR-22-3p/DAPK2	upregulated	MIAT knockdown reduced cardiomyocyte apoptosis and improved left ventricular function through downregulating DAPK2 expression by sponging miR-22-3p	[77]
Neat1	STZ-induced diabetic mice model combined with a high-fat/sugar diet and HG-treated primary culture of neonatal cardiomyocytes	miR-140-5p/HDAC4	upregulated	activation of Neat1/miR-140-5p/HDAC4 axis attenuated diabetes-induced cardiomyocyte apoptosis	[79]
PVT1	HG-treated AC16 cardiomyocytes	miR-23a-3p/CASP10	upregulated	PVT1 facilitated high glucose-induced cardiomyocyte apoptosis through the miR-23a-3p/CASP10 axis	[80]
HOTAIR	human myocardial biopsies and serum samples, HG-treated AC16 cardiomyocytes	PI3K/Akt pathway	downregulated	HOTAIR overexpression increased the viability of cardiomyocytes through activation of the PI3K/Akt pathway	[84]
TINCR	Human myocardial biopsies and serum samples, HG-treated AC16 cardiomyocytes		downregulated	TINCR overexpression inhibited apoptosis of HG-treated cardiomyocytes	[85]
H19	STZ-induced diabetic rat model and HG-treated primary culture of neonatal cardiomyocytes	miR-675	downregulated	H19 induced mitochondria-mediated apoptosis by targeting miR-675/ VDAC1	[86]

**Table 2:** LncRNAs participating in the regulation of myocardial autophagy and pyroptosis in DCM.

LncRNAs	Experimental Model	Target Genes	Expression	Mechanism Involved	References
**Autophagy**					
GAS5	STZ-induced diabetic rat model and HG-treated H9c2 cells	miR-221-3p/P27	downregulated	GAS5 reversed histopathological changes and ameliorated myocardial function via facilitating myocardial autophagy by targeting mir-221-3p/p27 axis	[95]
Neat1	STZ induced diabetic rat model and HG-treated primary culture of neonatal cardiomyocytes	Foxo1	upregulated	Neat 1 promoted cardiomyocyte autophagy by up-regulating Foxo1 expression to increase hypoxia-reoxygenation injury	[97]
DCRF	STZ-induced diabetic rat model and HG-treated primary culture of neonatal cardiomyocytes	miR-551b-5p/PCDH17	upregulated	DCRF knockdown improved cardiac function and suppressed autophagy activation by targeting miR-551b-5p/PCDH17 axis	[98]
**Pyroptosis**					
KCNQ1OT1	Human serum samples, STZ-induced diabetic mice model, and HG-treated primary culture of neonatal cardiomyocytes			silencing KCNQ1OT1 alleviated cardiac pyroptosis by targeting miR-214-3p and caspase-1	[100,101]
MIAT	Human serum samples, STZ-induced diabetic mice model, and HG-treated primary culture of neonatal cardiomyocytes	miR-214-3p	upregulated	MIAT knockdown ameliorated cardiac pyroptosis by targeting miR-214-3p/ CASP1 axis	[102]
MALAT1	HG-treated H9c2 cells	miR-141-3p	upregulated	MALAT1 targeted miR-141-3p to promote HG-induced H9C2 cardiomyocyte pyroptosis	[103]
TINCR	STZ-induced diabetic rat model and HG-treated H9c2 cells	NLRP3	upregulated	TINCR aggravated pyroptosis through regulating NLRP3 by increasing its mRNA stability	[104]
GAS5	STZ-induced diabetic mice model and HG-treated cardiac muscle cell line (HL-1 cells)	miR-34b-3p/AHR	downregulated	GAS5 repressed NLRP3 inflammasome activation-mediated pyroptosis by sponging miR-34b-3p and enhancing AHR expression	[105]
